# Craniofacial and oral alterations in patients with Neurofibromatosis 1

**DOI:** 10.1186/s13023-018-0881-8

**Published:** 2018-08-09

**Authors:** Vivian Visnapuu, Sirkku Peltonen, Lotta Alivuotila, Risto-Pekka Happonen, Juha Peltonen

**Affiliations:** 1 Department of Cell Biology and Anatomy, Institute of Biomedicine, University of Turku, Kiinamyllynkatu 10, 20520 Turku, Finland; 2 Department of Dermatology, University of Turku and Turku University Hospital, TE6, Hämeentie 11, P O Box 52, FI-20521 Turku, Finland; 3 Department of Oral and Maxillofacial Surgery, University of Turku, Lemminkäisenkatu 2, 20520 Turku, Finland

**Keywords:** Neurofibromatosis 1, Oral soft tissue, Craniofacial alteration, Periapical Cemental dysplasia, Wide Mandibular Canal, Tooth Developoment, Dental age, Speech

## Abstract

Neurofibromatosis type 1 (NF1) is one of the most common inherited syndromes. The literature on craniofacial alterations associated with NF1 has been limited and partially contradictory. This review is based on literature search and the results of the clinical study “Craniofacial and Oral Alterations and Speech in patients with Neurofibromatosis 1”, carried out at the University of Turku and Turku University Hospital, Finland in 2006-2012. By the end of 2012, a total of 110 NF1 patients, 54 female and 56 male patients, were examined.

A part of our results confirms pre-existing understanding, a part is contradictory to previous considerations based mainly on case reports, and some are entirely novel. Specifically, our results confirmed that enlargement the mandibular canal is the most common abnormality of the mandible in patients with NF1. It should be noted, however, that this finding does not require treatment. Caries was not a major problem. In fact, it was less frequent in NF1 patients compared to reference population. These findings abrogate some previous perceptions. Novel findings of our project include periapical cemental dysplasia in females; short jaws, a finding which usually does not affect bite; and immunohistological analysis of oral mucosal abnormalities. Pioneering study on speech showed that various deviations were very common: As many as 94% of the participants showed some alterations.

To conclude, the awareness of craniofacial alterations common in NF1would help avoiding unnecessary and even harmful involvement, e.g. of periapical cemental dysplasia or enlarged mandibular canal which do not require treatment.

## Background

Neurofibromatosis type 1 (NF1) is a neurocutanous-skeletal syndrome caused by mutations in the *NF1* tumor suppressor gene located at 17q11.2 [1]. NF1 is one of the most common rare diseases with a birth incidence of ∼1:2000, and prevalence of ~ 1:2000–1:3000 [2, 3]. The inheritance follows an autosomal dominant trait with highly variable phenotypic expression [4, 5]. Neurofibromatosis 2 (NF2) is caused by mutations in the *NF2* gene on chromosome 22. Bilateral schwannomas of the eight-cranial nerve are the hallmark of NF2 [6]. The incidence of NF2 has been estimated to be 1 in 40,000 [6].

The diagnosis of NF1 is based on criteria outlined by the National Institute of Health Consensus Development Conference in 1987 [7]. Cutaneous neurofibromas and café au lait spots are the hallmarks of NF1 in skin [4, 8]. Larger neurofibromas grow deeper along peripheral nerves, and manifest as plexiform neurofibromas, which may form large and disfiguring tumor masses in the craniofacial region, trunk, and extremities [8]. Plexiform neurofibromas have a risk of malignant transformation [9]. Pilocytic astrocytomas of the optic nerve are the most common NF1-related tumors in the central nervous system [4].

Oral involvement has been reported to occur in 3.4–92% of adult patients, and in about 40% of children with NF1 [10–15]. We believe that these divergent numbers reflect paucity of research on this important field.

The current review is based on the project “Craniofacial and Oral Alteration and Speech in patients with Neurofibromatosis 1”, carried out at the University of Turku and Turku University Hospital in 2006–2012 [16–22]. Selected previously unpublished findings are discussed and pertinent literature is reviewed.

## Methods

A total of 110 patients with NF1, 54 female patients aged 3–68 years and 56 male patients aged 8–73 years, were included in our study and clinically examined by the same clinician (V.V., DDS). Orthopantomograms, cephalograms and periapical radiographs were taken and used to analyze dental age, dental caries, cephalometry and periapical cemental dysplasia, and other alterations in bony structures of jaws. Soft tissue tumors were operated and immunohistochmically characterized. Speech features were characterized and recorded.

To cover the previous literature on craniofacial and oral alterations in NF1, PubMed/MedLine (National Library of Medicine, Washington, DC), Google-Scholar and Scopus databases were searched for words neurofibromatosis 1 and: dental age, caries, periapical cemental dysplasia (PCD), wide mandibular canal, oral soft tissue, and speech from 1976 up to and including November 2017. Titles and summaries of the search results were screened for relevant studies, and full texts of articles chosen were retrieved. In addition to our studies [16–22], the search revealed three reviews [23–25], five case reports [26–30] and six original retrospective studies [10, 11, 13, 15, 31, 32].

## Results and discussion

### Dental age divergence in patients with NF1

The dental age is the result of dental maturation. Dental development is less affected by environmental insults than the skeletal maturation [31] and shows less variation between individuals than the development of the long bones. For that reason, the analysis of dental development is more accurate for the estimation of chronological age than the analysis of long bones [33, 34]. Aberration of the dental maturation may result in a situation where the observed dental age differs from the chronological dental age. To our knowledge, dental age has not previously been reported in NF1. In our study, 34 patients were evaluated for the timing of dental maturation based on x-rays. The results showed that dental age was unaffected in patients with NF1 up to the age of 17 years [23], (Table 1). The mean dental age for boys with NF1 was then held up by 0.32 years and for girls with NF1 it was brought forward by 0.02 years compared with Finnish norms [35]. Thus, our results do not explain the findings of Lammert et al. who have previously reported early eruption of the first primary teeth in children with NF1 compared with their unaffected siblings and with a normal control population [36].

**Table 1:** Conclusions of craniofacial findings in NF1 [17–22]

Dental age [22]	
No statistical difference compared to general population	
Caries (DMFT; decayed, filled and missing teeth) [20]	
Lower DMFT index in patients with NF1 < 35 years	
Caries is not feature of NF1	
Radiographical findings [16, 17, 21]	
Periapical cemental dysplasia (PCD)	
Bone deformities caused by plexiform neurofibromas	
Enlarged mandibular canal	
Retrognathic mandible and maxilla, and short cranial base	
Oral soft tissue alterations [18]	
Found in 37% of patients with NF1	
Overgrowth of gingival soft tissue in 28% of patients with NF1	
Prominent lingual papillae in 40% of patients with NF1	
Mucosal tumors, most of which are neurofibromas	
Speech characteristics [19]	
Voice quality alteration (eg. nasal voice, aberrant speech rhythm) in 35% of adults with NF1, and in 55% of children with NF1	

### Dental caries in patiens with NF1

Bardellini et al. reported in their case control study that children with NF1 showed poorer oral hygiene conditions in comparison to the control group in Italy [37]. Previous reports from Canada have suggested increased amount of caries in NF1 patients [38, 39]. These retrospective studies were based on questionnaires sent to families having at least one member with NF1 [38]. The results showed that dental caries was more often reported by siblings with NF1 as compared to siblings without NF1 in these families [38].

We believe that the diagnosis of caries can only be based on an examination by professional clinician and carried out in an appropriate setting. The dental health status was assessed in 110 Finnish patients with NF1 in a clinical study [20]. The results demonstrated that among individuals < 35 years of age, NF1 patients in fact presented lower rate of caries than has been reported for the reference population [18]. The reference population consisted of two large Finnish national cohorts: The one with 861,700 persons covered most children under 18 years of age within the public health care between years 1970 and 2000 [35], and the other one which was a random sample of 8028 persons of adults over 30 years of age [40]. A general notion was that the mean numbers of DMFT (decay-missing-filled-teeth) increased gradually by age category. In the category of 30–34 years, the mean values were lower in the NF1 patients than in the reference group. In the two oldest age categories (45–54 and 65+), there were no differences between the NF1 patients and the reference group in relation to the numbers of DMF-teeth [20]. Caries is due to bacterial infection. The reason for lower caries incidence in NF patients in Finland may be that oral health care and primary caries prevention are carried out more strictly in NF patients than in general population. The results suggest that NF1 as such does not cause susceptibility to caries (Table 1). A strict professional caries prevention is advised because gingival enlargements and motoric clumsiness may hamper the maintaining patient’s oral health and our results in agreement with recently published study of Friedrich et al. [41].

### Periapical cemental dysplasia in patients with NF1

**Fig. 1:**
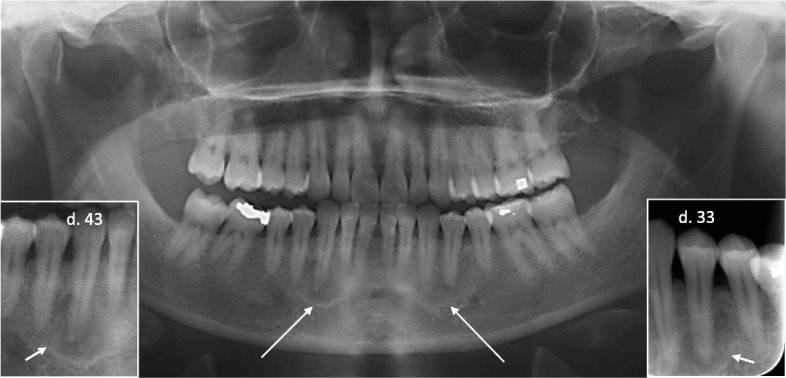
Orthopantomogram and two intraoral radiographs (insets) of a 35-year-old woman with NF1 shows periradicular radiolucencies with intralesional calcification around vital teeth number 33 and 43 (arrows) suggesting the mature stage of periapical cemental dysplasia

Radiographic images taken in our study revealed a novel NF1 related manifestation, periapical cemental dysplasia [17], (Table 1). Teeth associated with radiolucent inflammatory periapical lesions (endodontic lesions) have necrotic pulps, and therefore do not respond to vitality tests. In contrast, lesions of non-pulpal (non-endodontic) origin usually do not affect the blood or nerve supply to the adjacent tooth pulp, and therefore, these teeth remain vital [42]. Pulp vitality tests and careful patient history are the most important factors in differentiating between endodontic and non-endodontic lesions. The latter does not require active therapy whereas in the former, root canal treatment is necessary. We reported radiolucent periapical lesions which were diagnosed as periapical cemental dysplasia (PCD) in vital mandibular teeth of patients with NF1 (Fig. 1). Interestingly, eight females out of 55 patients with NF1 in our series had PCD [16]. None of the male patients or children showed similar findings. To avoid unnecessary treatments, it is important that PCD of NF1 patients is not confused with periapical findings caused by endodontic pathoses [16]. In fact, some of our patients had undergone root canal treatments on unsymptomatic incisors suggesting that PCD had been misdiagnosed for endodontic lesions. The pathogenesis of periapical cemental dysplasia remains unknown, but we speculate that the various bone malformations in NF1 may share a common cellular etiology. It should also be noted that PCD is the first apparent sexual dimorphism described to date in NF1.

### Special radiographical findings in jaws in patients with NF 1

A high prevalence of oral and radiographic findings among NF1 patients have been reported by Shapiro et al. in 1984 [13]. The most common jaw malformations are intrabony lesions, such as wide mandibular canals and enlarged mandibular foramina [13], (Table 1). These findings have been further affirmed in studies by D’ Ambrosio 1988 [26], Kaplan et al. in 1994 [32] and Lee et al. 1996 [43]. In a clinical study on 48 NF1 patients, Friedrich et al. [15] reported 26 patients with plexiform neurofibromas originating from the branches of the trigeminal nerve. These 26 patients had alterations of tooth position, deformities of the adjacent bones and malocclusion. In the other 22 patients with NF1, malformations of the alveolar ridge were absent and individual oral symptoms were rarely found or were mild [15].

**Fig. 2:**
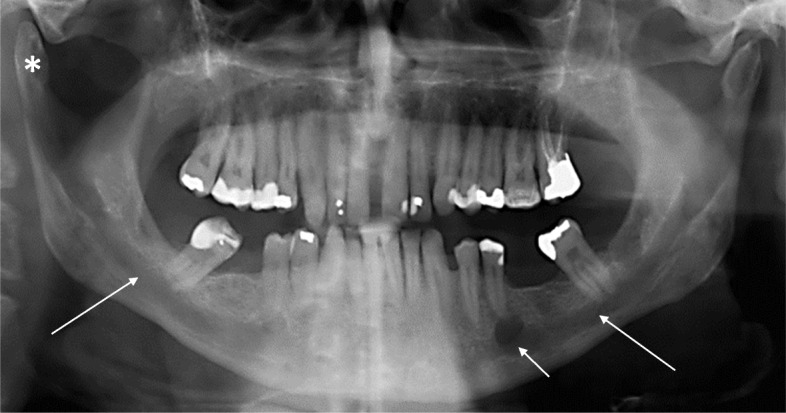
Orthopantomogram of a 55-year-old man with NF1. The mandibular canals (long arrows) on both sides of the mandible are widened in their full length and the mental foramen (short arrow) on the left side is enlarged. Note also the elongated right mandibular condyle (*)

Our study confirmed that enlarged mandibular foramen and mandibular canal (Fig. 2) are the most common findings of the mandible in patients with NF1 [21]. The association of enlarged mandibular foramen has been reported to be independent of tumor mass [13]. In patients without plexiform neurofibroma, bilateral widening of the mandibular canal was seen in 11, and unilateral widening in 10 out of 96 patients. In patients with plexiform neurofibroma, widening of the mandibular canal was seen only on the side affected by the tumor in 5 of 6 patients. The enlargement varied from slight widening of the canal to 2-fold in diameter. Also, an irregular border of the mandibular canal was frequently seen as a sign of involvement of the nerve. It is logical to assume that the enlargement of the mandibular canal can be caused by the overgrowth of the mandibular nerve, although no direct evidence on the nerve structure is available [21].

Widening of the mandibular canal and other bone defects associated with the canal are easy to detect on routine panoramic radiologic images, and do not require further attention or treatment. It is however important that oral and maxillofacial surgeons and dentists recognize these abnormalities as common features of NF1. Thus, widening of mandibular canal should raise a suspicion of NF1 in cases who may miss the diagnosis of NF1.

### Craniofacial characteristics in patients with NF1

Skeletal lesions are not only considered pathognomonic of NF1, but also comprise one of the important diagnostic criterias [44–46]. In addition to long bone lesions, osseous manifestations of NF1 are seen in the facial skeleton, which include sphenoid wing and orbital dysplasia, maxillary and mandibular deformities, and rarely temporomandibular joint deformities [46].

We used cephalograms to investigate craniofacial skeletal malformations in patients with NF1 [17]. The results showed that patients with NF1 typically had a short mandible, maxilla, and cranial base compared with healthy controls, irrespective of age, but the results were statistically significant only in adults [18], (Table 1). The length of the mandible, the maxilla and the cranial base correlated with the height of patients under 19 yr. of age, but this correlation was absent in adult patients. The mandibular ramus was shorter in patients with NF1 compared with controls, but this was only detected in adult patients.

**Fig. 3:**
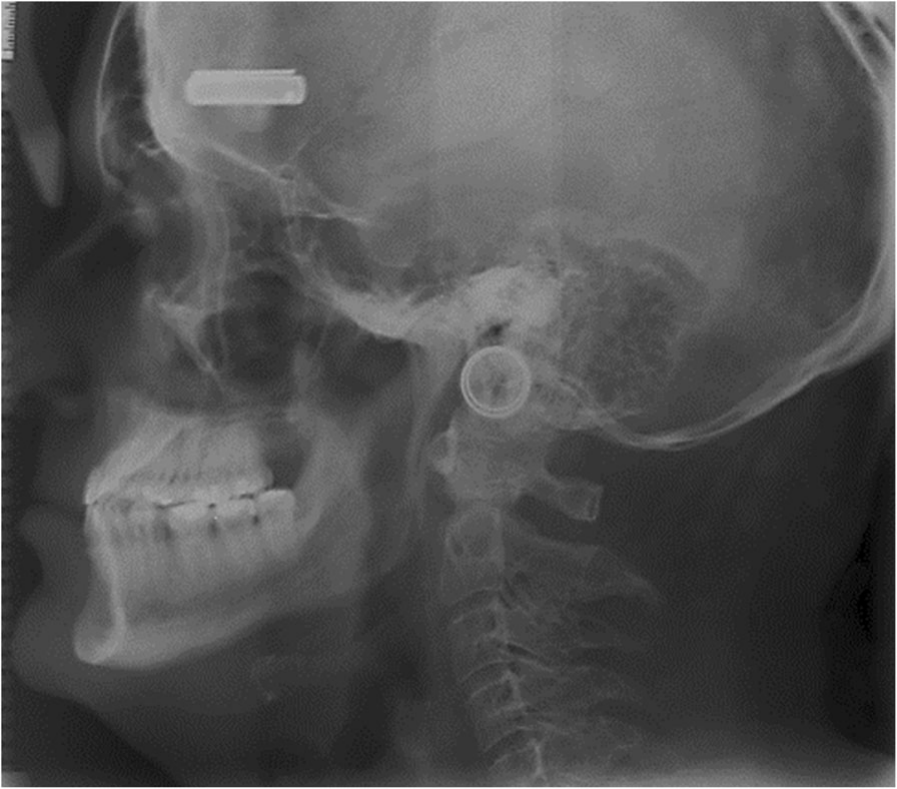
Lateral skull radiograph of a 53-year-old man with NF1 shows marked maxillary retrognatia

The maxilla was short in 75% on NF1 patients (Fig. 3), and it was also often retrognathic when compared with controls. This was determined by measuring the sella–nasion–point A angle, which was, in general, 3° less than in controls. The short anterior cranial base was statistically significant only in adulthood. The distance between the porion and the pterygoid was short in most of male adult patients with NF1 compared with healthy controls [17].

To conclude, facial bones in most patients with NF1 are short in the anteroposterior direction, but the anteroposterior diameter of the whole skull is greater than in persons without NF1 [17]. Cung et al. [47] have also reported that shorter maxilla, mandible, cranial base, and diminished facial height are characteristic for adults but not for children. The recent study of Luna et al. [48] confirm our results of short mandible, maxilla and skull base in patients with NF1, which is important knowledge to dentists and physicians.

Jaws and the cranial base are largely derived from the neural crest, and NF1 is considered as a pathosis of neural crest, or a neurocristopathy [49–52]. Craniofacial dysmorphism is also associated with other developmental disorders of the Ras-pathway, including Legius, Noonan, Costello, cardio-facio-cutaneous, and LEOPARD syndromes. Interestingly, mouse model of the Legius syndrome (Spred-1 knockout mice) has short jaws [24, 53, 54]. Thus, the findings in human and mouse suggest that Ras pathway is essential for the normal growth of craniofacial structure.

In addition to genetic factors, skeletal malformations could be due to local factors provoked by the presence of tumors.

### Oral soft tissue alterations in patients with NF1

**Fig. 4:**
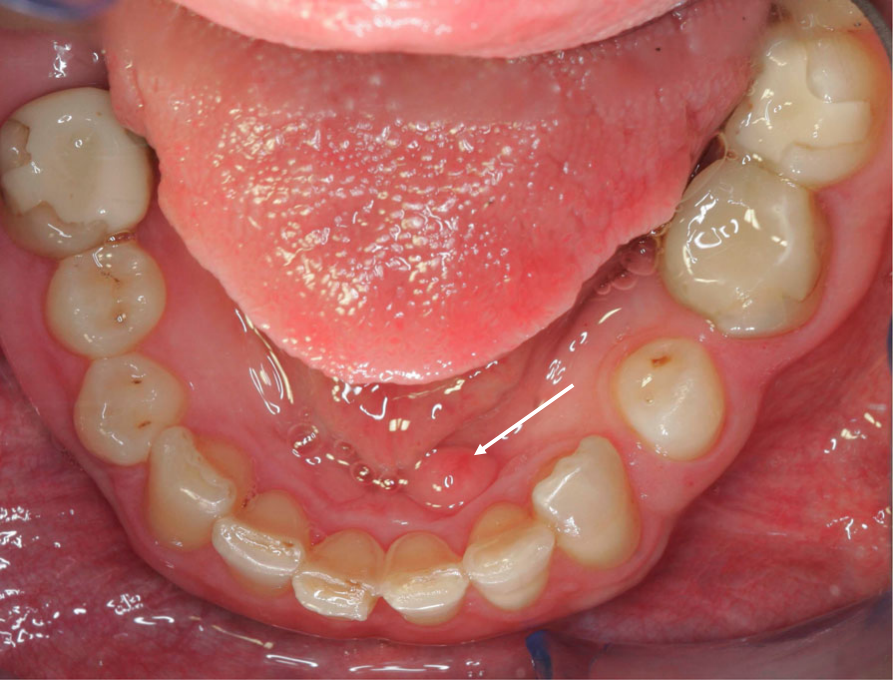
A solitary mucosal neurofibroma (arrow) behind frontal teeth of lower jaw in a 58-year-old woman with NF1. Note also irregularities on both sides of the edge of the tongue arising suspicion of soft tissue overgrowth

**Fig. 5:**
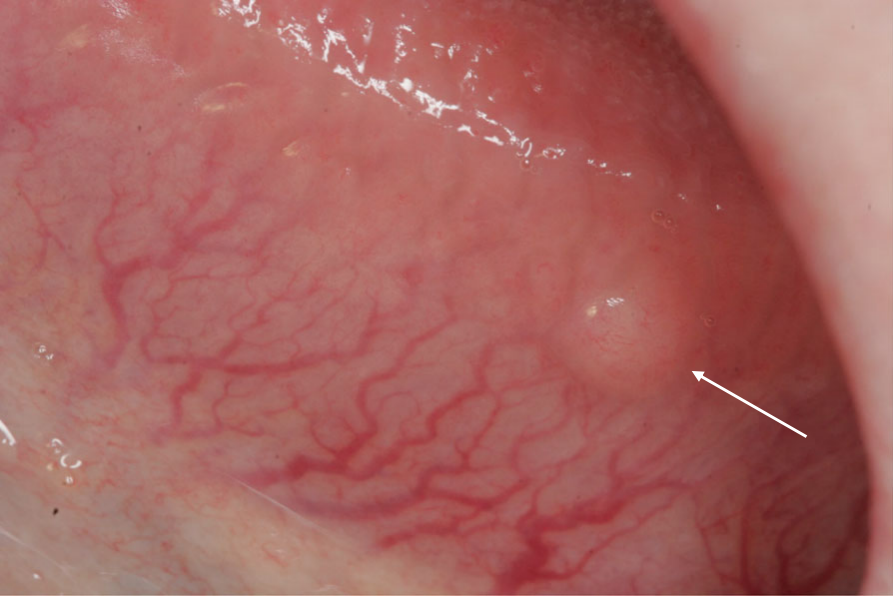
Mucosal neurofibroma (arrow) on dorsal tongue in 30-year-old woman with NF1

Oral soft tissue manifestations in NF1 have been reported in a few full-length papers [13, 18, 25, 26, 55–57], including a review of literature [55]. In our study [18], the most common findings were prominent lingual papillae, overgrowth of gingival soft tissue, and mucosal tumors (Fig. 4). Of all NF1 patients, 74% had oral soft tissue alterations, and they were equally common in both sexes [19], (Table 1). Oral mucosal tumors were present in 37% of patients, and the most common location was the tongue (Fig. 5). These results were in accordance with previous studies [15, 18, 56].

The frequency of gingival soft tissue overgrowth increased according to age. Discrete oral mucosal neurofibromas typically started to grow during puberty which is in accordance with cutaneous neurofibromas. The frequency of oral tumors in children (0–12 years) was 8%; in adolescents (13–18 years) 14%; and 39% in adults [18]. Also, prominent lingual papillae were more common in adolescents (36% of patients) and adults (45%) than in children (28%). The soft tissue lesions classified as gingival overgrowths in our study were more frequent than those reported in the literature. However, some of these changes may in fact represent neurofibromas in analogy to those described by Cunha et al. [58] and García de Marcos et al. [59].

The plexiform neurofibromas apparently affecting the second branch of the fifth cranial nerve were detectable on the buccal mucosa, the soft tissue on the mandible and the tongue [15, 18]. According to the literature, the histology and immunohistology of the oral plexiform neurofibromas are indistinguishable from plexiform neurofibromas affecting other anatomical locations [55–57].

Results of our study and previous studies show that the clinical inspection and palpation are not sufficient for the diagnosis of oral neurofibroma even if the patient had NF1 [13, 18, 25]. We noted also that routine histology either was not constantly sufficient for definite diagnosis since the histological structure of mucosal neurofibromas was more heterogeneous than that of cutaneous neurofibromas. Immunohistochemistry proved to be highly useful in the analyses of solitary tumors. Specifically, a panel of immunoreactions including S100, type IV collagen, CD34, and neurofilament or neuron-specific tubulin (TUBB3) demonstrated the presence of neural involvement in the fibromatous tumors [18]. Toluidine blue staining visualized mast cells within the oral mucosal neurofibromas, which is in analogy to findings on cutaneous neurofibromas [57, 60, 61].

Soft tissue changes were most common in maxillary tuberosities [15, 18, 56]. Although oral mucosal neurofibromas were frequent in NF1 patients, there were only few of them per patient. This differs from skin, where the number of cutaneous neurofibromas per anatomical area may be high.

Oral soft tissue tumors may affect speech [19] and may cause discomfort for which reason the clinical oral examination of these patients is recommended. Troubling discrete intraoral tumors can be excised, but the treatment of plexiform neurofibromas is more complicated. A thorough histopathological analysis supported by immunohistochemistry is essential for the correct diagnosis of these oral soft tissue growths.

### Speech features in patients with NF1

Many patients with NF1 have a communication disorder [62], including deviations in speech and language. For instance, reading and writing problems are not uncommon [63, 64]. Lorch et al. [63] reported that patients may suffer from motor dyspraxia, which induces mispronunciations, problems in sequencing sounds, and deviant prosody. Previous findings on seven patients with NF1 were found to present speech alterations affecting mainly the sounds /r/ and /s/ [65, 66]. Persons with NF1 may have abnormal speaking rate, volume, pitch, hoarseness, and/or hypernasality [64]. Thompson et al. [67] observed significant differences in the speech and language instruments in the group of children with NF1 compared to the validated controls. Furthermore, 68% of the children with NF 1 exhibited delays in speech and/or language, 32% demonstrated delays in articulation, 37% percent demonstrated delays in receptive language, and 37% exhibited delays in expressive language. A total of 16% of the children exhibited a voice disorder, and 42% were judged to have a resonance problem [67].

Although previous studies report a variety of characteristics in the speech of patients with NF1, a detailed study describing the physical aspects of speech has not been published to our knowledge.

Our results of 62 patients show that deviations in the speech of patients with NF1 are common [19]. The results suggest motoric dysfunction of different anatomical parts of the vocal tract, namely the vocal cords, the velum and the tongue. Persons with NF1 typically have problems in pitch regulation, resulting in monotone speech. According to our findings, patients with NF1 often have deviant phonation, namely strained, breathy, creaky or hoarse voice quality, as well as nasal voice. Similar problems have also been reported in previous studies with smaller numbers of participants [64]. Some patients with NF1 have either missing harmonics or the entire harmonic structure is scrambled in their voice [19]. Deviations in phonation may well attest for our subjective perception that many patients (especially male) tend to sound alike.

Many of the patients in this study tended to speak loud and fast, which may contribute to the impression of NF1-typical speech [20], (Table 1). Those who speak fast generally articulate less clearly and may reduce or delete sounds and syllables. Our results agree with Lorch et al. [63] who have noticed patients with NF1 to have abnormal rate, volume, pitch, and articulation. We reported also articulation errors to be common, especially in the sibilant /s/. Finnish language has only one sibilant speech sound, and varying manifestations of /s/ should not cause interpretation problems [19]. However, a speaker of a language that distinguishes different types of /s/−sounds, for example, alveolar and postalveolar sibilants, would likely interpret a patient’s abnormal /s/ as postalveolar sibilant (as in English word sheet). This is agreeing with Zorzi and Assencio-Ferreira [65], who report that two of their seven patients substituted the alveolar phoneme /s/ with the postalveolar kind. The range and cause of the phenomenon will require a more thorough investigation.

We observed several different types of dysfluent speech but did not detect stuttering in any of the 62 patients examined, which is contrary to conclusions of previous reports [64, 68]. In contrast, sequencing tasks proved to be extremely challenging for patients with NF1, probably reflecting problems in the central rather than the peripheral level of motor control. If true, our results support the conclusions of Lorch et al. [63] who speculated that speech problems in patients with NF1 are due to both motor coordination problems and difficulties in planning and sequencing complex tasks.

The speech problems in patients with NF1 appear similar in different languages. However, phonological systems vary in different languages, and some languages may be more challenging than others.

The reasons behind speech aberrations in NF1 patients are poorly understood. Factors related to brain function [69] and functional disturbancies of peripheral nerves apparently contribute to the problems in speech production. In addition, oral overgrowths and tumors may disturb speech production. Furthermore, cognitional difficulties [70] and learning difficulties may affect the speech production.

Anyone in close contact with patients with NF1, for instance educators and physicians should be made aware of the fact that patients with NF1 may have trouble in communication, and that the lack of finesse in their self-expression is not a sign of unsocial attitude [68, 69]. To improve the patient’s quality of life and to provide more effective speech therapy for those in needs, the underlying causes of speech deviations in NF1 need to be discovered [71, 72]. Our study [19] highlights selected components of speech which are often difficult for patients with NF1 and are apparent targets for customized speech therapy.

### Other empirical craniofacial findings in patients with NF1

Abundant salivary secretion was observed in clinical examination of patients with NF1, although the absolute amounts of saliva were not measured. This may also in part contribute to the presence of less caries in NF1 patients compared to the general population although gingival enlargemets seen in NF patients hamper maintaining of oral hygiene.

Another notion in clinical examination was sensitive emetic reflex of patients with NF1. This may be partly explained by the short cranial base (distance between) the nasion and the sella compared with controls which was noted in 75% of the adult patients with NF1 [17].

## Conclusion

The article reviews the up-to-date literature on the craniofacial and oral alterations in patients with NF1. The most common radiological findings include enlargement of mandibular canal and mental foramen. Clinical hallmarks for suspicion of NF1 consist of intraoral neurofibromas and overgrowth of gingival soft tissue. Due to common aberrations in speech production (eg. abnormal rate, volume, pitch, articulation errors in the sibilant /s/ sounds) the voice of many NF1 patients sound alike (“NF1 speech”). All dentists and oral and maxillofacial surgeons should be aware of these features arising suspicion of NF1 and refer the patient to a thorough medical and genetic examination. Early diagnosis of NF1 is extremely important for young patients. Orthodontic treatment can be utilized to prevent the development bite anomalies common in NF1 patients. Furthermore, early involvement of speech pathologists in the multidisciplinary treatment group of NF1 patients is important.

## Abbreviations

NF1: Neurofibromatosis 1
NF2: Neurofibromatosis 2
PCD: Periapical cemental dysplasia
PubMed/MedLine: National Library of Medicine, Washington, DC
TUBB3: Neuron-specific tubulin
